# Comparative Phenotypic and Transcriptomic Analyses Provide Novel Insights into the Molecular Mechanism of Seed Germination in Response to Low Temperature Stress in Alfalfa

**DOI:** 10.3390/ijms25137244

**Published:** 2024-06-30

**Authors:** Zhao Zhang, Yanzhen Lv, Qingying Sun, Xingjie Yao, Huifang Yan

**Affiliations:** 1 College of Grassland Science, Qingdao Agricultural University, Qingdao 266109, China; 18366690350@163.com (Z.Z.); lyz15610475121@163.com (Y.L.); 15949823182@163.com (Q.S.); 15806930125@163.com (X.Y.); 2 Key Laboratory of National Forestry and Grassland Administration on Grassland Resources and Ecology in the Yellow River Delta, Qingdao 266109, China; 3 Qingdao Key Laboratory of Specialty Plant Germplasm Innovation and Utilization in Saline Soils of Coastal Beach, Qingdao 266109, China

**Keywords:** low temperature stress, alfalfa, transcriptome analysis, seed germination, glutathione metabolism

## Abstract

Low temperature is the most common abiotic factor that usually occurs during the seed germination of alfalfa (*Medicago sativa* L.). However, the potential regulatory mechanisms involved in alfalfa seed germination under low temperature stress are still ambiguous. Therefore, to determine the relevant key genes and pathways, the phenotypic and transcriptomic analyses of low-temperature sensitive (Instict) and low-temperature tolerant (Sardi10) alfalfa were conducted at 6 and 15 h of seed germination under normal (20 °C) and low (10 °C) temperature conditions. Germination phenotypic results showed that Sardi10 had the strongest germination ability under low temperatures, which was manifested by the higher germination-related indicators. Further transcriptome analysis indicated that differentially expressed genes were mainly enriched in galactose metabolism and carbon metabolism pathways, which were the most commonly enriched in two alfalfa genotypes. Additionally, fatty acid metabolism and glutathione metabolism pathways were preferably enriched in Sardi10 alfalfa. The Weighted Gene Co-Expression Network Analysis (WGCNA) suggested that genes were closely related to galactose metabolism, fatty acid metabolism, and glutathione metabolism in Sardi10 alfalfa at the module with the highest correlation (6 h of germination under low temperature). Finally, qRT-PCR analysis further validated the related genes involved in the above pathways, which might play crucial roles in regulating seed germination of alfalfa under low temperature conditions. These findings provide new insights into the molecular mechanisms of seed germination underlying the low temperature stress in alfalfa.

## 1. Introduction

Alfalfa (*Medicago sativa* L.), a perennial herbaceous plant in the leguminous family, has a natural biological nitrogen fixation function, excellent nutritional quality, and a high biomass yield, therefore having a variety of beneficial effects on soil coverage, livestock, and human health [1]. In China, alfalfa has been grown for more than 2000 years, and it is primarily cultivated and widely distributed in northern regions [2]. Normally, alfalfa is sown in early spring or late autumn. However, with the increasingly severe global climate and environmental situations, low temperatures have occurred frequently and always impose cold stress on plants. In particular, its sudden occurrence during the seed germination stage after sowing, due to the unstable temperature in early spring in China, results in low and non-uniform seedling emergence, abnormal seedling morphogenesis, and unsuccessful establishment of plants, which further restricts alfalfa productivity [3]. Therefore, temperature is crucial for the early planting of alfalfa, and improving the seed germination ability under low temperature conditions through modern molecular breeding approaches could be beneficial for its production.

Low temperature is a most common abiotic factor in plant survival, which seriously restricts the geographical distribution, growth and development, and yield formation of plants [4]. Once the external environmental temperature drops below the minimum threshold required for seed germination, it can cause irreversible damage to cells and tissues, normally even leading to germination failure [5]. It is documented that low temperatures restrain seed germination by affecting various physiological and metabolic events, including imbalances of ions and osmolates, disrupted stability of the cell membrane system, and secondary oxidative stress, which in turn further impede cell respiration and normal metabolism during the seed germination process [6]. Particularly, oxidative damage is the most common insult to cellular homeostasis in seed physiology. Under low temperatures, reactive oxygen species (ROS) are excessively produced and the dynamic balance is broken, leading to enzymatic activities like superoxide dismutase (SOD), lipid peroxidation, DNA damage, protein denaturation, carbohydrate oxidation, and decreased enzyme activity [7,8]. Studies have shown that lipids are the vital part of cell membranes, which are the main sites of low-temperature damage caused by the peroxidation of unsaturated fatty acids [9]. To resist and balance the damage caused by low temperatures, plants have developed multiple protection strategies to activate the defense mechanism, such as synthesizing detoxification antioxidants and enzymes, increasing the content of intracellular osmotic substances such as betaine, proline, and soluble sugars, and accumulating secondary metabolites [10,11]. Among them, the triggered antioxidant system is very important and mainly regulated by antioxidant enzymes and ascorbic acid-glutathione (AsA-GSH) cycle, involving SOD, peroxidase (POD), catalase (CAT), glutathione peroxidase (GPX), ascorbate peroxidase (APX), glutathione reductase (GR), monodehydroascorbate reductase (MDHAR), and dehydroascorbate reductase (DHAR) [12]. Reduced glutathione (GSH) is a non-enzymatic antioxidant that is essential for maintaining cellular redox homeostasis [13]. A previous report in maize (*Zea mays* L.) showed that the AsA-GSH cycle played a key role in eliminating excessive accumulated ROS, thus reducing oxidative damage induced by low temperatures [14]. In brief, low temperatures affect seed germination through various physiological and biochemical processes and limit the transition of seeds to normal seedlings and plants, thereby reducing plant production and yield.

Numerous studies have indicated that low temperatures cause extensive alterations at the molecular level, which are controlled by a complex and finely-tuned regulatory network and associated encoding genes [15]. In order to enhance the adaptation and tolerance of plants to low temperatures, multiple genes are activated and jointly encode stress-responsive proteins, including kinases, transcription factors, and other functional genes [16]. For instance, in maize, low temperature tolerance genes like *ZmICE1*, *ZmbZIP68*, *ZmRR1*, and *ZmDREB1* have been cloned and functionally studied [17,18,19]. Previous studies in alfalfa also demonstrated that some genes were involved in altering the cold stress tolerance of plants, such as autophagy-related genes *MsATG13* [20] and calmodulin-like proteins *MsCML46* [21] and *MsCML10* [22], through modulating autophagy, antioxidant level, signal transduction, and K^+^/Na^+^ balance. Furthermore, gene regulation modes of the PYR/PYL/RCAR-PP2C-SnRK2-ABF/AREB pathway [23] and ICE-CBF-CORs pathway [24], the most well-understood mechanisms, are proven to be two main ways for plants to respond to low temperature stress. Therefore, the reported findings have increased our understanding of the genetic mechanisms underlying low temperature tolerance in plants.

Seed germination is the most critical stage in a plant’s lifecycle and is also the most sensitive to external environmental factors. It usually starts with water uptake by mature dry seeds and terminates with the protrusion of the seed envelope [25]. During the germination process, seeds must quickly shift from a relatively quiescent status to an active metabolic status and undergo a series of complex molecular and physiological alterations [26]. Based on water absorption characteristics, seed germination consists of three phases [27]. Phase I, a rapid water absorption period, plays a vital role during seed germination, in which seed respiration is rapidly enhanced and provides energy for reactivating the necessary physiological processes in the subsequent phases [28]. As well, phase II (a plateau of water uptake) is also a critical stage because gene expression and metabolic activity are largely enhanced [29]. Thus, these two phases lay the important substances (carbohydrates, amino acids, nucleic acids, etc.) and energy foundation for successful seed germination and subsequent normal seedling morphogenesis. In fact, seed germination is a process by which seeds gradually lose their dehydration tolerance, which can be re-induced within a developmental window during early germination [30]. It has been reported that different types of histone post-translational modifications are involved in the epigenetic regulation of seed germination and dehydration tolerance [31]. In some plant species, such as maize [32], cotton (*Gossypium hirsutum* L.) [33], and hybrid rapeseeds (*Brassica napus* L.) [34], it has been reported that there were genotypic differences in seed germination characteristics and tolerance ability to low temperatures among various germplasms. Over the past few years, although alfalfa’s responses to low temperature stress have attracted much attention from scientists, most studies have mainly analyzed the detrimental effects of low temperature on the phenotypic traits of seedlings, roots, and mature leaves [35,36,37]. To date, however, the key genes and metabolic pathways involved in alfalfa seed germination under low temperature stress still remain largely unknown. Therefore, it is necessary to elucidate the underlying molecular and genetic mechanisms of seed germination under low temperature stress in alfalfa, which will provide a good foundation for its subsequent cultivation and harvest.

Transcriptome analysis has become a powerful tool for mining genes, which plays an increasingly important role in understanding the relationship between gene expression differences and complex phenotypes. It has been extensively conducted to explore the molecular mechanisms of seed germination responses to low temperatures in various plants, such as maize [38], cotton [33], hybrid rapeseeds [34], and rice (*Oryza sativa* L.) [39]. Shen et al. [33] analyzed the responses of seed germination to low temperature in two cotton varieties with different cold tolerances, and it indicated that the main differences were centered at glycolysis/gluconeogenesis, tricarboxylic acid cycle, and glyoxylate cycle. So far, however, the relevant genes and pathways have only been partially revealed and still remain largely unknown. For the important perennial forage, the existing studies on the low temperature responses of alfalfa seed germination are primarily limited to the phenotypic and physiological levels; the key genes and regulatory networks that are identified at the transcriptomic level are still unclear. Hence, revealing the molecular basis and excavating the key genes regulating alfalfa seed germination at low temperatures are extremely urgent.

Nowadays, research on the responses of alfalfa seed germination to low temperatures mostly focuses on the comparison and evaluation of low temperature tolerance in different cultivars and the screening of key indicators [40,41,42], while the specific mechanisms involved in this process are relatively little studied. Therefore, it is important to explain the underlying mechanisms of alfalfa seed germination at low temperatures. In this study, two alfalfa cultivars were selected, namely Instict (a low temperature-sensitive genotype) and Sardi10 (a low temperature-tolerant genotype), that exhibited significant differences in seed germination responses to low temperatures. Subsequently, transcriptome analyses were conducted to reveal the involved genes and pathways and to explore the potential molecular mechanisms of alfalfa seed germination under low temperatures. These findings will not only contribute to providing valuable insights into the molecular mechanisms underlying the low temperature tolerance of alfalfa but also lay a theoretical foundation for the breeding of its new cultivars.

## 2. Results

### 2.1. Morphological Differences in Seed Germination of Two Alfalfa Cultivars under Low Temperature Stress

**Figure 1:**
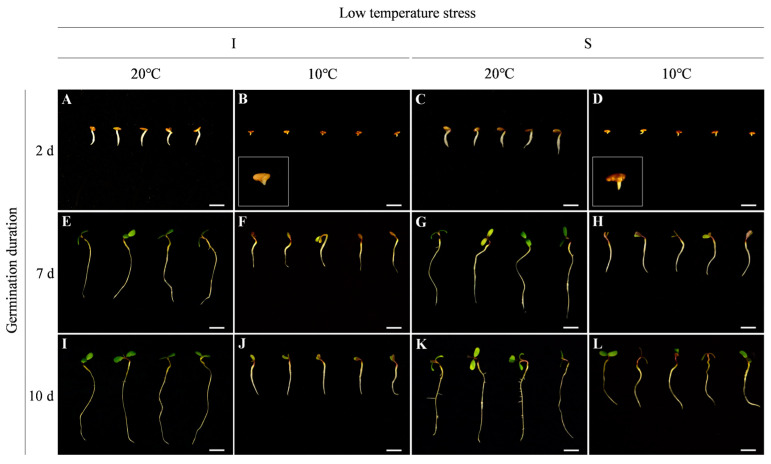
Phenotypic differences in seed germination between two alfalfa cultivars under low temperature stress. (**A**,**B**) Instict cultivar under 20 °C and 10 °C after 2 days of germination. (**C**,**D**) Sardi10 cultivar under 20 °C and 10 °C after 2 days of germination. (**E**,**F**) Instict cultivar under 20 °C and 10 °C after 7 days of germination. (**G**,**H**) Sardi10 cultivar under 20 °C and 10 °C after 7 days of germination. (**I**,**J**) Instict cultivar under 20 °C and 10 °C after 10 days of germination. (**K**,**L**) Sardi10 cultivar under 20 °C and 10 °C after 10 days of germination. In the bottom left corner of (**B**,**D**), the amplified image is for a clearer observation of the radicle differences after 2 days of germination. I and S, respectively, mean Instict and Sardi10. Scale bar = 1 cm.

After 2 days of germination, there was no significant difference in radicle length between two cultivars at 20 °C, but the radicle protrusion and growth status of Sardi10 were better than those of Instict at 10 °C (Figure 1A–D). Furthermore, as seeds continued to germinate until 7 and 10 days, seedlings that had completed morphogenesis did not differ significantly between two cultivars at 20 °C, while the radicle length of Sardi10 was significantly longer than that of Instict at 10 °C (Figure 1E–L).

**Figure 2:**
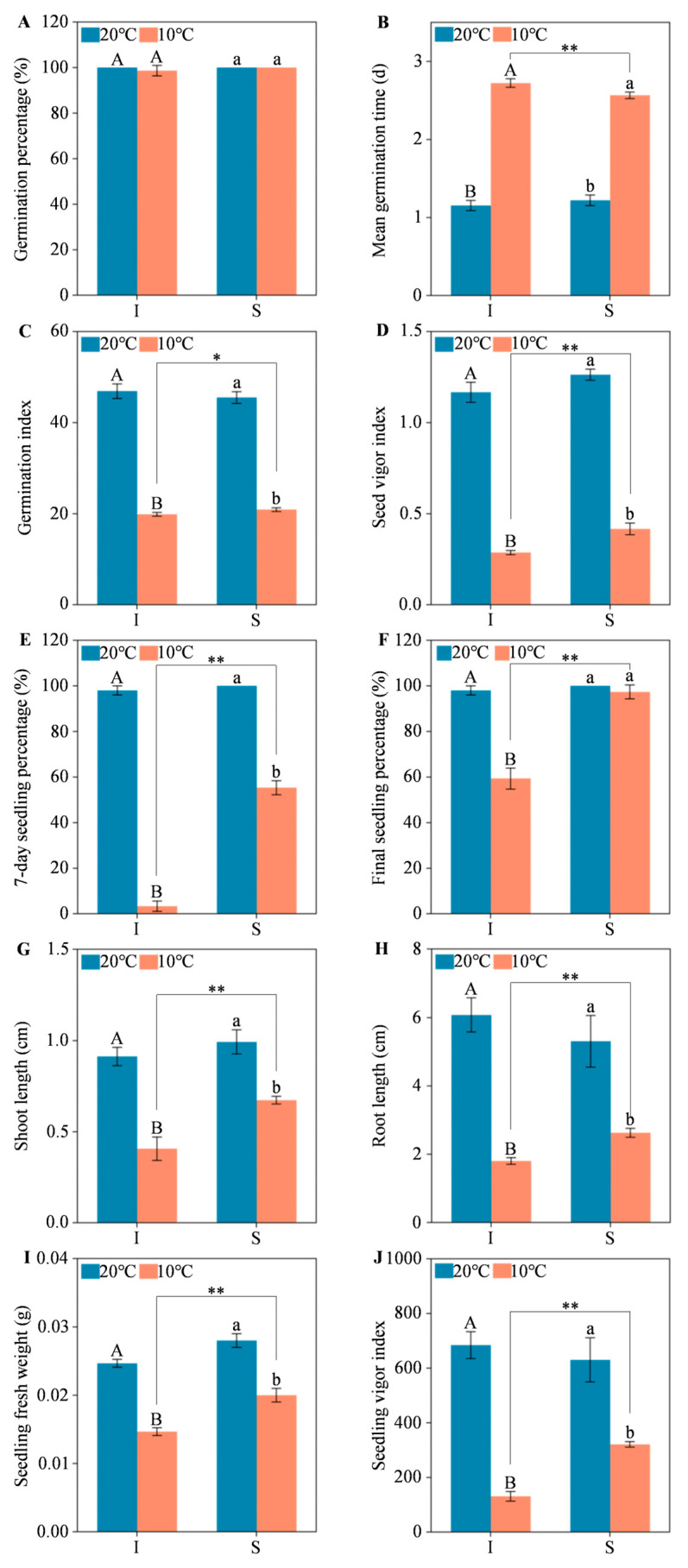
Quantitative analysis of seed germination-related indicators between two alfalfa cultivars under low temperature stress. (**A**) Germination percentage. (**B**) Mean germination time. (**C**) Germination index. (**D**) Seed vigor index. (**E**) 7-day seedling percentage. (**F**) Final seedling percentage. (**G**) Shoot length. (**H**) Root length. (**I**) Seedling fresh weight. (**J**) Seedling vigor index. Different uppercase and lowercase letters indicated significant differences between temperatures in Instict and Sardi10, respectively, at a level of *p* < 0.05. The * and ** indicated, respectively, a significant difference (*p* < 0.05) and an extremely significant difference (*p* < 0.01) between Instict and Sardi10 under 10 °C, based on the Student’s *t*-test. Vertical bar represents the standard error of three replicates. I and S, respectively, mean Instict and Sardi10.

For both two alfalfa cultivars, low temperature stress did not result in a significant decrease in germination percentage, but it led to a significant increase in mean germination time and significant decreases in germination index, seed vigor index, 7-day seedling percentage, final seedling percentage, shoot length, root length, seedling fresh weight, and seedling vigor index, except for the final seedling percentage of Sardi10 without difference. It was also found that the phenotypic indicators did not show significant differences between Instict and Sardi10 under 20 °C, while they presented significant differences under 10 °C. Moreover, except for the mean germination time with a lower value, some other indicators of Sardi10 were significantly higher than those of Instict under 10 °C, such as the germination index, seed vigor index, 7-day seedling percentage, final seedling percentage, shoot length, root length, seedling fresh weight, and seedling vigor index (Figure 2). These above findings illustrated that Sardi10 had a significantly stronger low-temperature tolerance and higher germination capacity than Instict.

**Figure 3:**
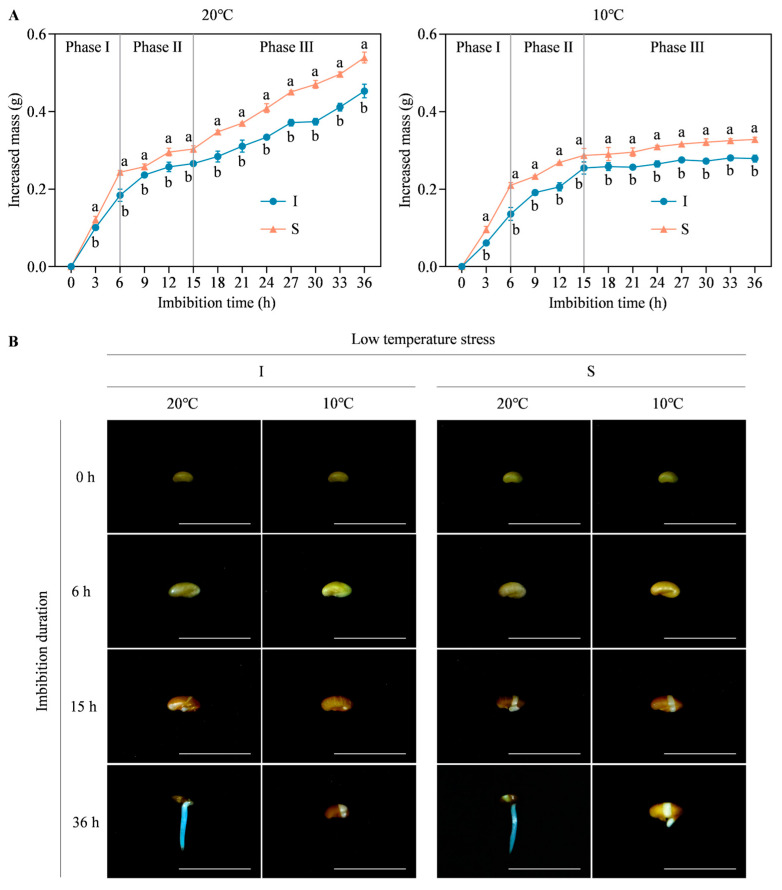
Establishment of imbibition curve during seed germination of two alfalfa cultivars under low temperature stress. (**A**) Dynamic changes of increased mass in imbibed seeds during germination. Different lowercase letters indicated significant differences between two alfalfa cultivars under 20 °C and 10 °C, respectively, at *p* < 0.05 level. Vertical bar represents the standard error of three replicates. (**B**) Seed imbibition phenotypes at early germination phases. Scale bar = 1 cm. I and S, respectively, mean Instict and Sardi10.

Based on the seed imbibition curve, it showed that the first 6 h was related to a rapidly increased mass (phase I), followed by a plateau of seed mass from 6 to 15 h (phase II), and then another rapid increase of seed mass from 15 to 36 h (phase III, Figure 3A). Meanwhile, seed imbibition phenotypes during germination phases revealed that two alfalfa genotypes showed different responses to low temperature stress (Figure 3B). Although Instict and Sardi10 seeds completed the physical imbibition within 6 h at 20 °C or 10 °C, the radicle expansion of Sardi10 seeds was slightly larger than that of Instict seeds at 10 °C. At 15 h, the seed coat of Sardi10 cracked, and the radicle was just about to break through the seed coat under both temperatures; however, Instict seed exposed its radicle under 20 °C but not 10 °C. These results indicated that Sardi10 seeds were more tolerant to low temperature stress than Instict seeds during the early germination phases. Nevertheless, it was conjectured that the subtle differences in imbibition phenotypes during early germination stages were affected by gene expression and physiological metabolic changes, which might be the important reason for the obvious differences in later germination and seedling growth. Therefore, 6 h (at phase I) and 15 h (at phase II) were identified as the early phases of alfalfa seed germination. Transcriptome analysis of Instict and Sardi10 imbibed seeds under 20 °C and 10 °C conditions was conducted to uncover the gene responses during the early germination stages of alfalfa seeds.

### 2.2. Overview of Transcriptomic Analysis

Transcriptome sequencing was performed, and a total of 1,055,156,980 raw reads were generated. After filtering out the low-quality data, 527,578,490 clean reads were obtained and used for assembly, generating 1,5792,710,2430 clean bases. The Q20 and Q30 base percentages were above 97.70% and 93.42%, respectively. The GC content ranged from 41.21–42.76%. Among clean reads, 76.70–82.02% were successfully mapped to the alfalfa genome, with an average mapping rate of 79.44%. Moreover, 72.05–76.87% of clean reads were uniquely mapped, with an average mapping rate of 74.64% (Supplementary Table S1). In addition, a total of 63,156 genes were identified, of which about 3.08–3.82% were highly expressed (FPKM ≥ 60) in all samples [43] (Supplementary Table S2). Overall, the above results indicated that the quality control of the sequencing data was excellent and could be used for subsequent analysis.

### 2.3. Identification of DEGs in Response to Low Temperature Stress

**Figure 4:**
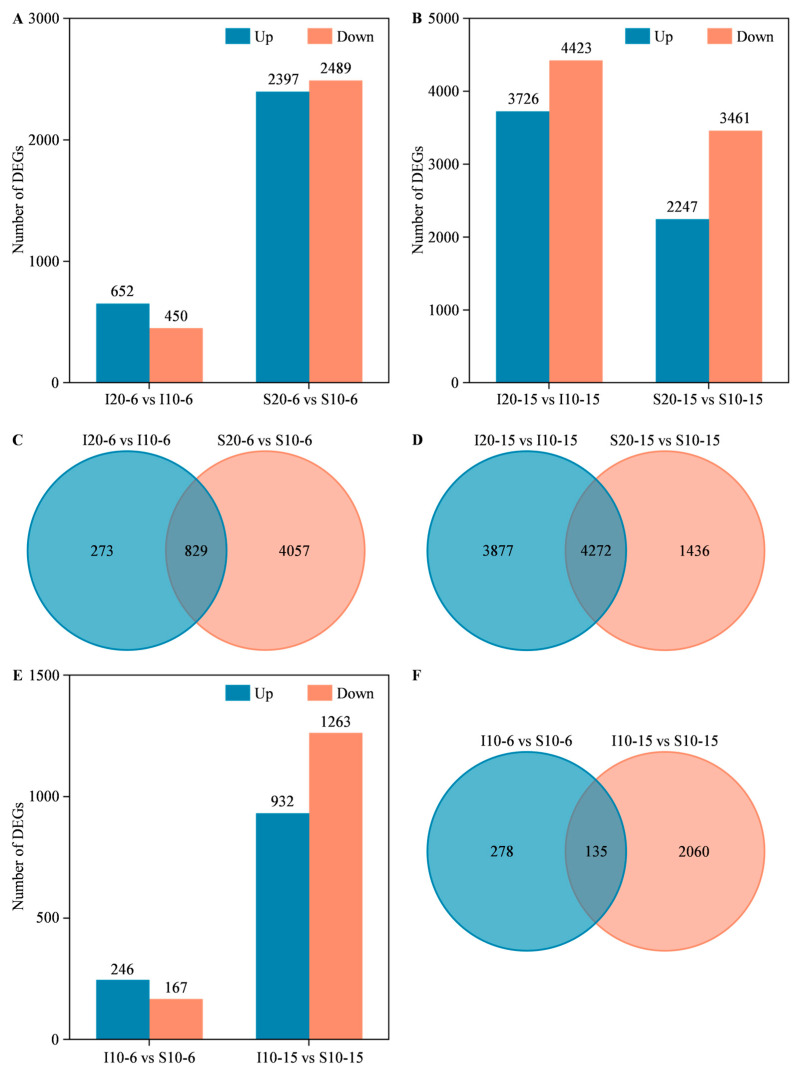
Profiles of differentially expressed genes (DEGs) associated with early seed germination of two alfalfa cultivars under low temperature stress. Number of up-regulated and down-regulated DEGs in paired comparisons between temperatures at (**A**) 6 h and (**B**) 15 h of seed germination. Venn diagram of DEGs in paired comparisons between temperatures at (**C**) 6 h and (**D**) 15 h of seed germination. (**E**) Number of up-regulated and down-regulated DEGs in paired comparisons between cultivars at 6 and 15 h of seed germination. (**F**) Venn diagram of DEGs in paired comparisons between cultivars at 6 and 15 h of seed germination. I and S, respectively, mean Instict and Sardi10.

Differential gene expressions in Instict and Sardi10 seeds in response to low temperature stress (10 °C) were compared with normal temperature (20 °C), respectively, at 6 and 15 h of germination. The number of up-regulated (log_2_FC ≥ 1 and FDR < 0.01) and down-regulated (log_2_FC ≤ −1 and FDR < 0.01) DEGs in each paired comparison was counted. It was found that, at 6 h of seed germination, 652 up-regulated and 450 down-regulated DEGs were identified in I20-6 vs. I10-6, while 2397 up-regulated and 2489 down-regulated DEGs were identified in S20-6 vs. S10-6, respectively (Figure 4A). Moreover, as seeds germinated for 15 h, more DEGs were identified. Concretely speaking, 3726 up-regulated and 4423 down-regulated DEGs were identified in I20-15 vs. I10-15, and 2247 up-regulated and 3461 down-regulated DEGs were identified in S20-15 vs. S10-15 (Figure 4B). Furthermore, a Venn diagram was used to present DEGs. Totally, 5159 DEGs were identified in two alfalfa cultivars at 6 h of seed germination, with 273 ones specifically in I20-6 vs. I10-6, 4057 ones specifically in S20-6 vs. S10-6, and 829 ones commonly in both I20-6 vs. I10-6 and S20-6 vs. S10-6 (Figure 4C). Meanwhile, with seed germination extending to 15 h, a total of 9585 DEGs were identified in two alfalfa cultivars, of which 3877 and 1436 ones were, respectively, specifically expressed in I20-15 vs. I10-15 and S20-15 vs. S10-15, and 4272 ones were commonly identified in both I20-15 vs. I10-15 and S20-15 vs. S10-15 (Figure 4D). Based on the above results, it indicated that Sardi10 had more DEGs than Instict at 6 h of seed germination under low temperature stress (Figure 4A,C). While the seed germinated for 15 h under low temperature stress, although the common DEGs shared by two cultivars increased, the total and specific DEGs in Sardi10 were less than in Instict (Figure 4B,D). Therefore, low temperature stress caused the differences in expression changes of genes related to early seed germination in two alfalfa cultivars.

In addition, differential gene expressions were also compared between two alfalfa cultivars under low temperature stress (10 °C). The specific DEGs in Sardi10 were detected at 6 and 15 h of seed germination under low temperature stress, which could be associated with the low temperature tolerance of Sardi10. Compared with Instict, 413 (246 up-regulated and 167 down-regulated) and 2195 (932 up-regulated and 1263 down-regulated) DEGs were confirmed in Sardi10, respectively, at 6 h (I10-6 vs. S10-6) and 15 h (I10-15 vs. S10-15) of germination (Figure 4E). Among all these DEGs, 278 and 2060 ones were specifically identified in Sardi10, respectively, at 6 and 15 h of seed germination under low temperature stress (Figure 4F, Supplementary Table S3). Hence, these findings suggested that there were differences in gene responses related to early seed germination between genotypes under low temperature stress.

### 2.4. GO Functional Enrichment Analysis

**Figure 5:**
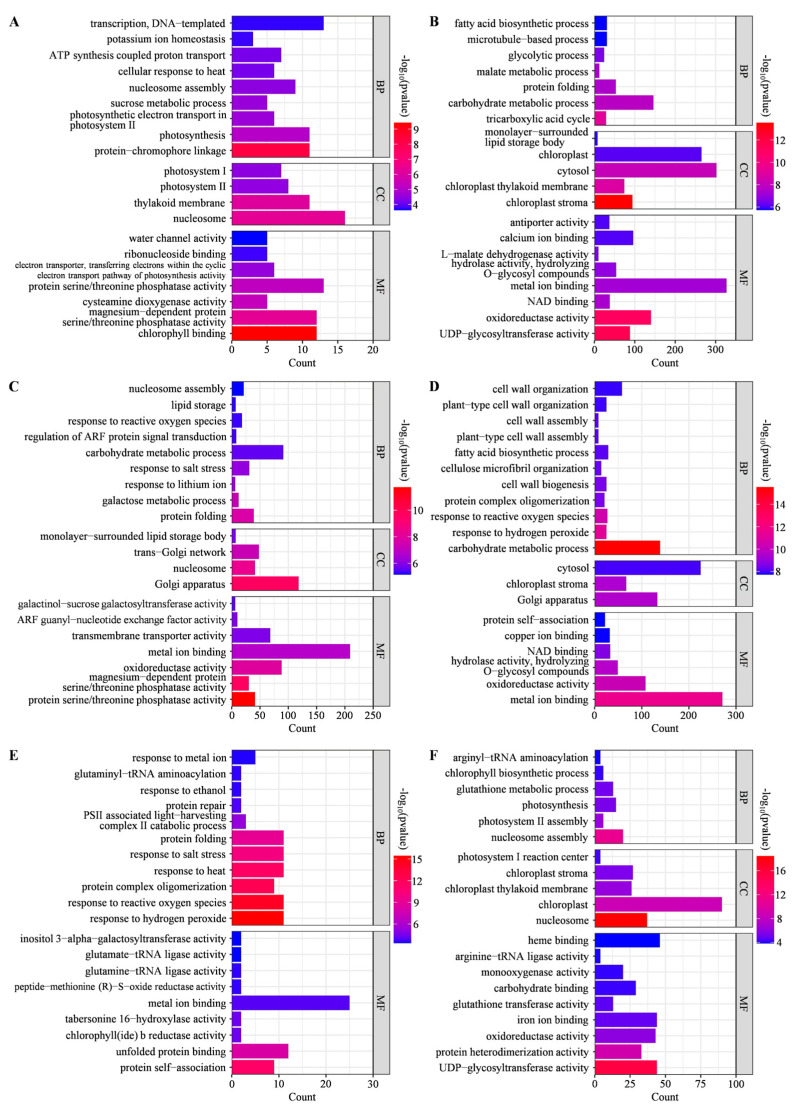
Top 20 enriched GO terms of DEGs involved in early seed germination of two alfalfa cultivars under low temperature stress. (**A**) I20-6 vs. I10-6. (**B**) I20-15 vs. I10-15. (**C**) S20-6 vs. S10-6. (**D**) S20-15 vs. S10-15. (**E**) I10-6 vs. S10-6. (**F**) I10-15 vs. S10-15. I and S, respectively, mean Instict and Sardi10.

GO enrichment of DEGs revealed the gene functions in response to low temperature stress in two alfalfa cultivars during early seed germination. Through the utilization of the GO database, DEGs were classified into three ontologies, including biological process (BP), cellular component (CC), and molecular function (MF) (Supplementary Table S4). Among all GO terms, the top 20 enriched ones were described in two groups of paired comparisons, namely, those between low and normal temperatures in each cultivar and those between two cultivars under low temperature stress. In Instict alfalfa, DEGs were highly enriched in several GO terms, such as protein-chromophore linkage, nucleosome, magnesium-dependent protein serine/threonine phosphatase activity, and etc. in I20-6 vs. I10-6 (Figure 5A), and tricarboxylic acid cycle, UDP-glycosyltransferase activity, oxidoreductase activity, and etc. in I20-15 vs. I10-15 (Figure 5B). However, DEGs in Sardi10 alfalfa were highly enriched in GO terms including protein folding, Golgi apparatus, protein serine/threonine phosphatase activity, etc. in S20-6 vs. S10-6 (Figure 5C), and carbohydrate metabolic process, response to hydrogen peroxide, response to reactive oxygen species, Golgi apparatus, oxidoreductase activity, etc. in S20-15 vs. S10-15 (Figure 5D).

Moreover, DEGs identified between two alfalfa cultivars were mainly enriched in GO terms of response to hydrogen peroxide, response to reactive oxygen species, protein complex oligomerization, response to heat, etc. in I10-6 vs. S10-6 (Figure 5E), while in I10-15 vs. S10-15, DEGs were mainly enriched in GO terms of glutathione metabolic process, nucleosome, UDP-glycosyltransferase activity, protein heterodimerization activity, etc. (Figure 5F). These above results indicated that there were partial differences between Instict and Sardi10, and the highly enriched GO terms might lead to differences in seed germination under low temperature stress.

### 2.5. KEGG Enrichment Analysis of DEGs

The KEGG enrichment analysis of DEGs was conducted to further determine the molecular mechanisms of alfalfa seed germination under low temperature stress. Totally, in Instict alfalfa, there were 112 and 134 KEGG pathways enriched in I20-6 vs. I10-6 and I20-15 vs. I10-15, while in Sardi10 alfalfa, 133 and 135 KEGG pathways were respectively enriched in S20-6 vs. S10-6 and S20-15 vs. S10-15. Moreover, for KEGG pathways between two cultivars, there were 82 and 117 ones enriched in I10-6 vs. S10-6 and I10-15 vs. S10-15 (Supplementary Table S5).

**Figure 6:**
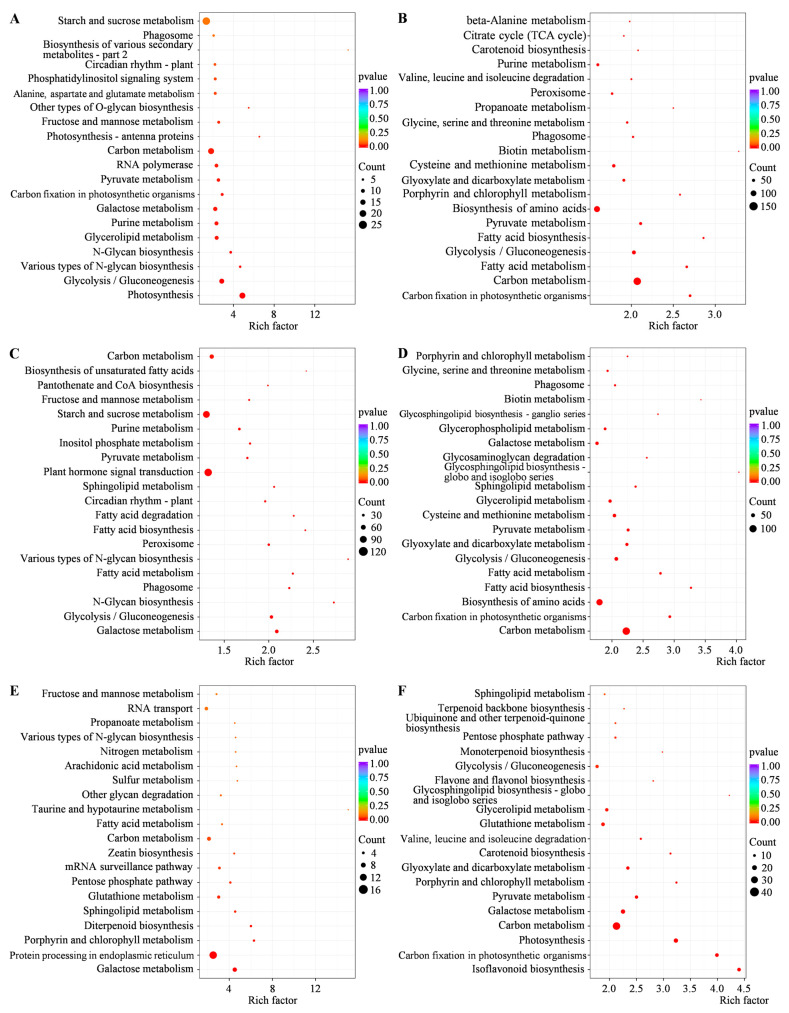
Top 20 enriched KEGG pathways of DEGs involved in early seed germination of two alfalfa cultivars under low temperature stress. (**A**) I20-6 vs. I10-6. (**B**) I20-15 vs. I10-15. (**C**) S20-6 vs. S10-6. (**D**) S20-15 vs. S10-15. (**E**) I10-6 vs. S10-6. (**F**) I10-15 vs. S10-15. I and S, respectively, mean Instict and Sardi10.

Among all these pathways, the top 20 ones were further presented (Figure 6). Notably, galactose metabolism and carbon metabolism pathways were significantly enriched and shared in all paired comparisons of I20-6 vs. I10-6, I20-15 vs. I10-15, S20-6 vs. S10-6, S20-15 vs. S10-15, I10-6 vs. S10-6, and I10-15 vs. S10-15 (Figure 6, Supplementary Table S5). It indicated that these two pathways might be the most important and fundamental ones for alfalfa seed germination in response to low temperature stress, regardless of the low temperature-sensitive or tolerant genotypes or the duration of low temperature stress.

In addition, according to KEGG enrichment analysis between two cultivars, pathways of sphingolipid metabolism, glutathione metabolism, and pentose phosphate pathway were significantly enriched in Sardi10 alfalfa, the low temperature-tolerant genotype, at both 6 and 15 h of germination at 10 °C (Figure 6E,F, Supplementary Table S5). Furthermore, the fatty acid metabolism pathway was also enriched in Sardi10 at 6 h of germination under 10 °C. Therefore, it was speculated that DEGs involved in the above pathways and the correspondingly regulated metabolic processes might be the key reasons why Sardi10 seeds had a significantly stronger low-temperature tolerance and higher germination capacity than Instict seeds.

### 2.6. Weighted Gene Co-Expression Network Analysis (WGCNA)

**Figure 7:**
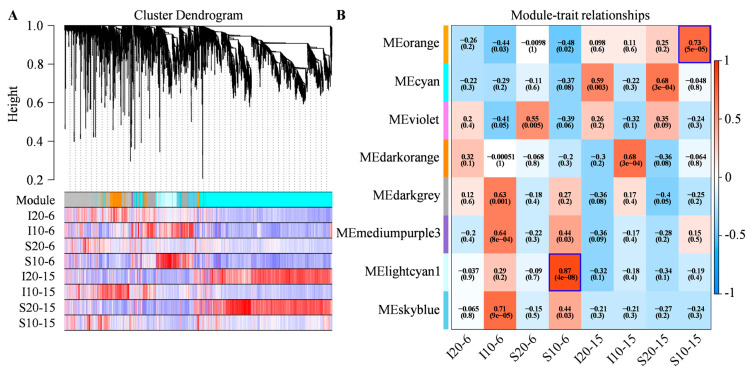
WGCNA analysis of two alfalfa cultivars during early seed germination under low temperature stress. (**A**) Hierarchical cluster tree showing the co-expression modules identified by WGCNA. In the “Module” color block, orange, cyan, violet, darkorange, darkgrey, mediumpurple3, lightcyan1, and skyblue represent different modules. (**B**) Correlations of the samples with WGCNA co-expression modules, i.e., relationships between modules (left) and traits (bottom). The red and blue backgrounds, respectively, represented the positive and negative correlations, with coefficient values and *p*-values.

A total of 5133 DEGs, after filtering out the low-abundance and low-variability genes, were used for WGCNA analysis to better analyze the molecular mechanisms of alfalfa seed germination under low temperature stress (Supplementary Table S6). Transcriptome data analysis was performed at each epoch for the WGCNA. The hierarchical clustering tree showed that each tree branch formed a module, and each leaf in the branch represented a gene (Figure 7A). Afterwards, the genes were identified according to their correlation with different germination phases of two alfalfa cultivars under normal and low temperatures. Based on WGCNA analysis, the 5133 DEGs were divided into eight modules, which were distinguished by different colors (Figure 7B, Supplementary Table S6). The module-trait relationships were different for each module that represented different germination phases of two alfalfa cultivars under normal and low temperatures. These modules contained genes regulated positively and negatively, and their expression levels varied depending on alfalfa cultivars, germination phases, and temperature treatments. To further uncover the most valuable pathways and possible mechanisms, two modules that were highly correlated with low temperature treatments of Sardi10 (the low temperature-tolerant genotype) were analyzed and explored: MElightcyan1 (r = 0.87, *p* = 4 × 10^−8^) in S10-6 and MEorange (r = 0.73, *p* = 5 × 10^−5^) in S10-15.

**Figure 8:**
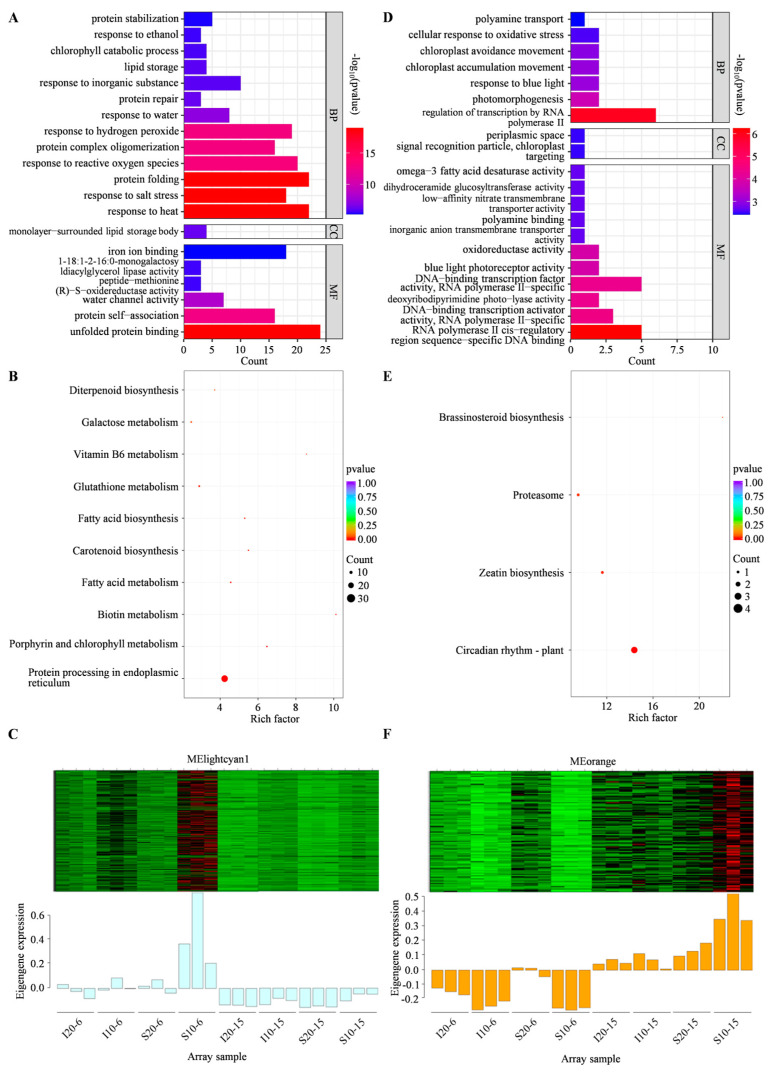
GO and KEGG analyses of DEGs in MElightcyan1 and MEorange modules identified by WGCNA. (**A**) Top 20 GO terms in MElightcyan1 module. (**B**) Significantly enriched KEGG pathways in MElightcyan1 module. (**C**) Eigengene expression in MElightcyan1 module at each treatment. The heatmap from red to green indicates eigengene expression from high to low, respectively. (**D**) Top 20 GO terms in MEorange module. (**E**) Significantly enriched KEGG pathways in MEorange module. (**F**) Eigengene expression in MEorange module at each treatment. The heatmap from red to green indicates eigengene expression from high to low, respectively.

Subsequently, to further explore the biological functions of DEGs in the above two modules identified by WGCNA, the GO and KEGG analyses were carried out. In MElightcyan1, GO terms of response to heat, monolayer-surrounded lipid storage body, unfolded protein binding, etc. were enriched, respectively, for BP, CC, and MF (Figure 8A). Meanwhile, KEGG pathways were enriched in fatty acid metabolism, glutathione metabolism, galactose metabolism, etc. (Figure 8B). The eigengene expressions of MElightcyan1 were most prominent in S10-6 (Figure 8C). In addition, MEorange module was highly correlated with S10-15. Genes for GO annotations mainly focused on regulation of transcription by RNA polymerase II, RNA polymerase II cis-regulatory region sequence-specific DNA binding, etc. (Figure 8D), and those of KEGG enrichment were circadian rhythm, zeatin biosynthesis, proteasome, and brassinosteroid biosynthesis (Figure 8E). The eigengenes of the MEorange module were mainly highly expressed in S10-15 (Figure 8F). Interestingly, it was found that the MElightcyan1 module related to S10-6 was highly enriched in KEGG annotation of fatty acid metabolism, glutathione metabolism, and galactose metabolism, which were also the enriched pathways at S10-6 identified by KEGG enrichment analysis of DEGs (Figure 6 and Figure 8B). Therefore, based on the comprehensive results of WGCNA and KEGG pathway analyses, galactose metabolism, carbon metabolism, fatty acid metabolism, and glutathione metabolism were determined as the key metabolic pathways for alfalfa seed germination in response to low temperature stress. The expressions of DEGs involved in the above pathways were conducted for an in-depth analysis and discussion.

### 2.7. The qRT-PCR Validation of DEGs in Alfalfa Seed Germination under Low Temperature Stress

**Figure 9:**
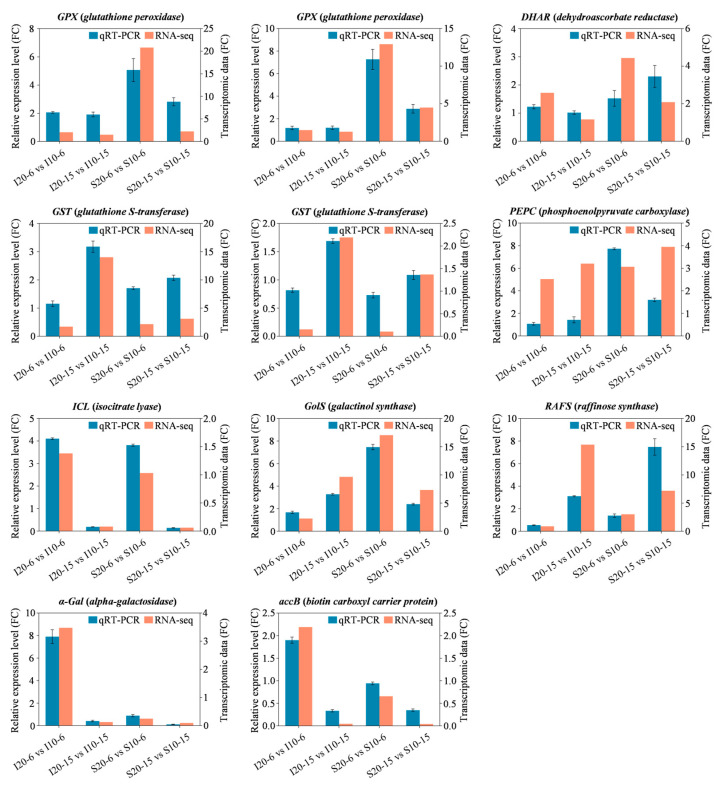
Expression analysis of the selected genes under low temperature stress by qRT-PCR.

To evaluate the accuracy of transcriptome data, eleven DEGs (*GPX*, *DHAR*, *GST*, *PEPC*, *ICL*, *GolS*, *RAFS*, *α-Gal*, and *accB*) were selected from galactose metabolism, carbon metabolism, fatty acid metabolism, and glutathione metabolism pathways for qRT-PCR analysis. The trend of relative expression levels of ten DEGs between qRT-PCR and transcriptome data was consistent (Figure 9), which verified the credibility of transcriptome sequencing.

## 3. Discussion

Seed germination, as the initial stage of plant growth and development, is not only determined by the biological properties of the seeds themselves but also affected by the surrounding environment. Generally, temperature, light, oxygen level, and soil moisture are the major factors that determine whether seeds can ultimately germinate and successfully emerge, among which temperature is the most crucial [44]. If seed germination, the most vulnerable stage, is subjected to low temperature stress, it will lead to serious germination or emergence failure, resulting in limited plant production and economic losses. Therefore, a higher germination performance of seeds under low temperature stress is a powerful guarantee for normal growth during the subsequent seedling stage [32]. In the present study, phenotypic and transcriptomic analyses of two cultivars of alfalfa seeds that germinated at 20 °C and 10 °C for 6 and 15 h were studied, which would provide new insights into candidate genes and pathways for seed germination under low temperature stress in alfalfa.

### 3.1. Differences in Low Temperature Responses of Two Alfalfa Cultivars during Seed Germination

Previous studies have reported that low temperatures have an inhibitory effect on alfalfa seed germination, reducing germination potential and germination index and prolonging mean germination time [37]. Furthermore, low temperatures also inhibited lateral root development, root and shoot growth of early seedlings, and decreased seedling survival with the extended duration of low temperatures [45]. In this study, low temperatures resulted in a significant decline in germination index, seed vigor index, 7-day seedling percentage, final seedling percentage, shoot length, root length, seedling fresh weight, and seedling vigor index for both alfalfa cultivars (Figure 2). Even so, there was no significant difference in the germination percentage of Instict and Sardi10 seeds under both normal and low temperature conditions (Figure 2A). Based on this, it indicated that low temperatures had no effect on the number of seeds whose radicle protruded through the seed coat but restricted the transition of seeds into seedlings after imbibition, thereby affecting the construction of normal seedlings. In addition, the other phenotypic indicators of Sardi10 alfalfa presented significantly higher than those of Instict alfalfa under low temperature stress, suggesting that Sardi10 had a significantly stronger low temperature tolerance and higher germination capacity than Instict.

Seed germination under low temperature stress involves various physiological events, including inhibition of soluble sugar accumulation, delay of starch degradation and consumption, and disruption of nucleic acid and protein synthesis, which is a complex process regulated by multiple genes and pathways [46]. In this study, although there were differences in seed germination phenotypes between two alfalfa cultivars under low temperature stress, the underlying mechanisms and key pathways involved in low temperature tolerance are still unclear. Therefore, to gain related insights, transcriptomic analysis was carried out, and some important genes and pathways were revealed. Notably, galactose metabolism and carbon metabolism pathways were significantly enriched by DEGs of both two alfalfa cultivars at 6 and 15 h of germination under low temperature stress, indicating that they might be the most important and fundamental pathways for seed germination in response to low temperature stress. Moreover, fatty acid metabolism and glutathione metabolism pathways were also significantly enriched in Sardi10 alfalfa during seed germination under low temperature stress, which might help explain its low temperature tolerance and high germination ability.

### 3.2. Galactose Metabolism Involved in Alfalfa Seed Germination in Response to Low Temperature Stress

**Figure 10:**
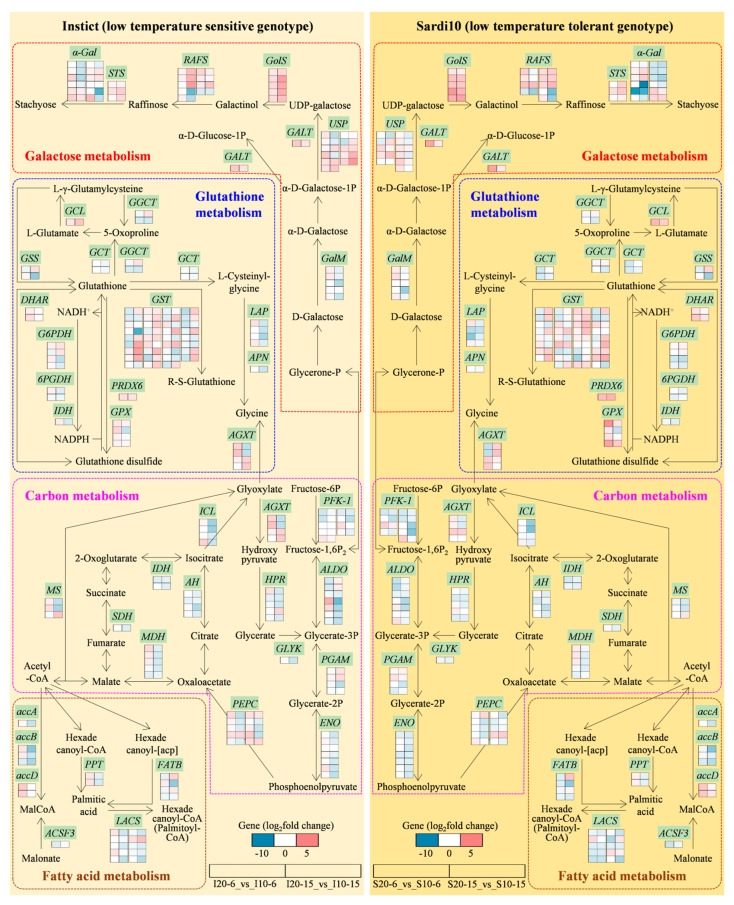
A putative model for low temperature tolerance of alfalfa during seed germination. Blue and red colors represent down-regulated and up-regulated DEGs by low temperature stress, respectively. Green boxes indicate gene-encoding enzymes. In galactose metabolism pathway: *α-Gal*, alpha-galactosidase; *STS*, stachyose synthase; *RAFS*, raffinose synthase; *GolS*, galactinol synthase; *USP*, UDP-sugar pyrophosphorylase; *GALT*, galactose-1-phosphate uridylyltransferase; *GalM*, aldose 1-epimerase. In carbon metabolism pathway: *MDH*, malate dehydrogenase; *SDH*, succinate dehydrogenase; *IDH*, isocitrate dehydrogenase; *AH*, aconitate hydratase; *PEPC*, phosphoenolpyruvate carboxylase; *ICL*, isocitrate lyase; *MS*, malate synthase; *PFK-1*, 6-phosphofructokinase 1; *HPR*, hydroxypyruvate reductase; *ALDO*, fructose-bisphosphate aldolase; *GLYK*, D-glycerate 3-kinase; *PGAM*, 2,3-bisphosphoglycerate-dependent phosphoglycerate mutase; *ENO*, enolase. In fatty acid metabolism pathway: *accA*, acetyl-CoA carboxylase; *accB*, biotin carboxyl carrier protein of acetyl-CoA carboxylase; *accD*, acetyl-CoA carboxylase carboxyl transferase subunit beta; *PPT*, palmitoyl-protein thioesterase; *FATB*, fatty acyl-ACP thioesterase B; *ACSF3*, malonyl-CoA/methylmalonyl-CoA synthetase; *LACS*, long-chain acyl-CoA synthetase. In glutathione metabolism pathway: *LAP*, leucyl aminopeptidase; *APN*, aminopeptidase N; *AGXT*, alanine-glyoxylate transaminase; *GGCT*, gamma-glutamylcyclotransferase; *GCT*, gamma-glutamyltranspeptidase; *GCL*, glutamate-cysteine ligase; *GSS*, glutathione synthetase; *G6PDH*, glucose-6-phosphate 1-dehydrogenase; *6PGDH*, 6-phosphogluconate dehydrogenase; *GPX*, glutathione peroxidase; *PRDX6*, peroxiredoxin 6; *DHAR*, dehydroascorbate reductase; *GST*, glutathione S-transferase.

Galactose is a common natural sugar found in several types of plant cell wall polymers that is also a component of the seed storage oligomers and their higher-order derivatives thereof, such as raffinose and stachyose [47]. The stored sugars in seeds are presumably carbon reserves for metabolism as well as storage molecules for germination and subsequent growth [47]. In this study, functional enrichment analysis of DEGs revealed that many genes associated with galactose metabolism were induced, indicating the crucial roles of sugars in regulating alfalfa seed germination in response to low temperature stress (Figure 10, Supplementary Table S7). During seed germination, raffinose and stachyose from the raffinose family oligosaccharides (RFOs) accumulate and are metabolized to sucrose by galactosidases, thereby liberating a large amount of galactose [48]. Raffinose synthase (RAFS) is the key enzyme for raffinose biosynthesis, catalyzing the formation of raffinose by transferring a galactosyl group of galactinol to sucrose [49]. Previous studies have reported that raffinose plays an important role in plant tolerance to drought [49], heat [50], chilling [51], and desiccation [52]. Herein, some *RAFS* were up-regulated when seeds germinated under low temperature for 6 h, especially in Sardi10, whereas several ones turned to downregulation with the prolonged germination duration to 15 h (Figure 10, Supplementary Table S7). It manifested that raffinose was primarily synthesized in the initial phase of germination, which contributed to providing more carbon reserves and energy for seeds to better adapt to low temperature stress during the germination process.

Galactinol is the precursor for RFOs synthesis, a D-galactose that binds to an inositol and is catalyzed by galactinol synthase (GolS) in plants [53]. Zhuo et al. [54] reported that cold-responsive *Arabidopsis AtGolS3* and *Medicago falcate MfGolS1* were induced by myo-inositol and resulted in plants with multiple tolerances to abiotic stresses. Moreover, overexpressed *CsGolS1* in cucumber (*Cucumis sativus* L.) enhanced the assimilate translocation efficiency and accelerated the growth rate of sink leaves, fruits, and whole plants under cold stress [55]. In this study, transcriptome analysis showed that *GolS* genes governing galactinol synthesis were upregulated, indicating that they might play vital roles in galactinol metabolism during alfalfa seed germination under low temperature stress (Figure 10, Supplementary Table S7). Furthermore, it was found that galactinol level was related to seed vigor in Arabidopsis, cabbage, and tomato, which in turn affected seed germinability and resistance to stresses [56]. Therefore, it was conjectured that Sardi10 alfalfa seeds might accumulate more galactinol in the early germination phases, thus having better resistance to low temperature stress than Instict alfalfa.

UDP-galactose is a substrate used for the synthesis of non-cellulosic polysaccharides and glycoproteins [57]. Although UDP-galactose is beneficial to plants, it has a certain range beyond which it will cause changes in polymers and glycoproteins, ultimately affecting cell wall architecture [47]. Aldose 1-epimerase, or mutarotase (GalM), is a key enzyme in carbohydrate metabolism, catalyzing the inter-conversion of alpha- and beta-anomers of hexose sugars, for instance, glucose and galactose [58]. UDP-sugar pyrophosphorylase (USP) catalyzes the conversion of various monosaccharide 1-phosphates to their respective UDP-sugars [59]. In this study, several DEGs related to UDP-galactose synthesis, including *GalM* and *USP,* were significantly downregulated, while *GolS* genes governing UDP-galactose degradation were upregulated (Figure 10, Supplementary Table S6). These findings indicated that low temperature stress repressed UDP-galactose synthesis and promoted UDP-galactose expenditure, thereby enhancing the production of more accessible galactinol for seed germination.

### 3.3. Carbon Metabolism Involved in Alfalfa Seed Germination in Response to Low Temperature Stress

Carbohydrates are the major storage reserves in seeds, which are produced and accumulated in specific tissues during plant growth and development [60]. Carbon metabolism is composed of glycolysis, the tricarboxylic acid (TCA) cycle, and metabolisms of polyols, organic acids, and fatty acids, and it is maintained by multiple regulatory events in cells to resist stresses under adverse conditions [61]. In the present study, functional enrichment analysis of DEGs revealed that many genes related to carbon metabolism were induced in both Instict and Sardi10 alfalfa seeds that germinated under low temperature stress, indicating the fundamental and crucial roles of carbohydrates in regulating seed germination (Figure 10, Supplementary Table S8). The TCA cycle is the key metabolic pathway for most organisms to harvest energy. It is generally believed to be responsible not only for the oxidization of respiratory substrates to drive ATP synthesis but also for providing carbon skeletons to anabolic processes and contributing to carbon-nitrogen interaction and biotic stress responses [62]. Multiple enzymes, such as malate dehydrogenase (MDH), succinate dehydrogenase (SDH), isocitrate dehydrogenase (IDH), and aconitate hydratase (AH), are involved in the TCA cycle and strictly regulate the interconversion of organic acids. Malate is one of the organic acids in the TCA cycle, which can protect plants against abiotic stress [63]. MDH, a multifunctional enzyme complex, catalyzes the reversible conversion of malate to oxaloacetate by utilizing NADH as a coenzyme. Results in winter rye (*Secale cereale* L.) showed that an increased malate content or MDH activity was induced by low temperatures, revealing that malate could serve as an additional sink for carbon assimilation at low temperatures [64]. Mitochondrial SDH, also known as the electron transport chain Complex II, plays a pivotal role in the TCA cycle and oxidative phosphorylation [65]. IDH is responsible for converting isocitrate to α-ketoglutarate in the TCA cycle and acts in glutamine synthesis [66]. In this study, *SDH*, *IDH*, and *AH* were downregulated in Instict and Sardi10 alfalfa seeds that germinated for 6 and 15 h under low temperature stress, while *MDH* was only upregulated at 6 h (Figure 10, Supplementary Table S8), suggesting that the reversible reaction between malate and oxaloacetate might be activated and the accumulated malate enabled alfalfa seeds to resist low temperature stress.

Phosphoenolpyruvate carboxylase (PEPC) is located at the core of carbon metabolism in plants and catalyzes the irreversible carboxylation of phosphoenolpyruvate to produce oxaloacetate for the TCA cycle [67]. Previous studies have shown that PEPC has multiple roles in plant growth and development, including seed development, seed germination, and enhanced tolerance to drought and salt stress [68,69,70]. Transcriptomic data in this study revealed that *PEPC* was upregulated at 6 h of germination under low temperature stress (Figure 10, Supplementary Table S8), suggesting that *PEPC* might perform a function at the initial stage of germination, thus providing the basic material for the TCA cycle. In addition, *PEPC* was proven to be related to lipid metabolism. In peanuts (*Arachis hypogaea* L.) [71] and upland cotton (*Gossypium hirsutum* L.) [72], the downregulation of *PEPC1* increased the oil accumulation and lipid content by 5.7%–10.3%. In this study, as seed germination duration extended to 15 h under low temperature stress, *PEPC* was partially downregulated (Figure 10, Supplementary Table S8). Therefore, it was speculated that a shift from carbon flowing into lipid metabolism might be promoted by inhibiting *PEPC* expression in alfalfa seed during germination under low temperature stress.

The glyoxylate cycle links carbohydrate metabolism and lipid metabolism during seed germination and early seedling growth, providing carbon intermediates for the biosynthesis of transportable sucrose and replenishing the TCA cycle under conditions where most intermediates are withdrawn for biosynthetic processes [73]. Previous studies in sugar beet (*Beta vulgaris* L.) showed that differential induction of enzymes involved in the glyoxylate cycle by stress appeared to be a physiological marker for distinguishing seedling vigor between cultivars [74]. Malate synthase (MS) is the key enzyme of the glyoxylate cycle and catalyzes the condensation of glyoxylate and acetyl-CoA to yield malate and CoA. Additionally, isocitrate lyase (ICL) is another key enzyme in the glyoxylate metabolic pathway [75]. In this study, only one *ICL* was upregulated in Instict seeds at 6 h of germination under low temperature, while at 15 h of germination, almost all *ICLs* and *MSs* were downregulated in both Instict and Sardi10 seeds (Figure 10, Supplementary Table S8).

### 3.4. Fatty Acid Metabolism Involved in Alfalfa Seed Germination in Response to Low Temperature Stress

Fatty acids are the important components in seeds, which serve as the major storage carriers for energy produced by photosynthesis in plants [76]. When seeds are subjected to adverse conditions during germination, the membranes are the first line of defense against biotic and abiotic stressors [77]. It has been reported that the fluidity and stability of membranes are closely related to low temperature tolerance in plants, and unsaturated fatty acids are generally considered to be more beneficial for maintaining membrane stability [78]. In the de novo biosynthesis of fatty acids, acetyl-CoA carboxylase (ACCase) catalyzes the first critical step, namely carboxylates acetyl-CoA to yield malonyl-CoA, the precursor for fatty acid synthesis and elongation, through ATP-dependent carboxylation of acetyl-CoA [79]. ACCase is composed of four distinct proteins, including biotin carboxyl carrier protein (accB, BCCP), biotin carboxylase (accC), and two proteins (accA and accD) catalyzing the carboxyltransferase partial reaction [80]. Previously, *BCCP* genes in *Brassica napus* L. and cotton were reported to show different expression patterns after cold and salt stress and played an important role in stress tolerance [81]. In this study, most DEGs associated with malonyl-CoA synthesis, including *accA*, *accB,* and *accD,* were upregulated at 6 h of germination under low temperature stress while being downregulated at 15 h (Figure 10, Supplementary Table S9). These findings indicated that malonyl-CoA might play a primary role in phase I and provide the energy necessary for subsequent seed germination. Malonate, the homolog of succinic acid, has been used as a competitive inhibitor of succinate dehydrogenase in the TCA cycle. It has been reported that malonate is toxic, but its low doses produce little or no toxicity [82]. Herein, *malonyl-CoA/methylmalonyl-CoA synthetase* (*ACSF3*) associated with malonate degradation was downregulated during alfalfa seed germination under low temperatures (Figure 10, Supplementary Table S9), speculating that malonate might be accumulated and produce toxicity for seed germination.

Long-chain acyl-CoA synthetase (LACS) catalyzes the synthesis of long-chain acyl-CoA from free fatty acid. In *Arabidopsis*, there are nine genes encoding *LACS* proteins, which play various roles in lipid metabolism [83]. Among them, *AtLACS2* was reported to enhance submergence tolerance by modulating cuticle permeability [84]. Moreover, ectopic expression of apple *MdLACS4* in *Arabidopsis* decreased epidermal permeability and water loss, thereby enhancing the resistance of transgenic plants to drought and salt stress [85]. However, *LACS* has been little reported in plant responses to low temperature stress. In this study, transcriptomic data revealed that *LACS* was partially induced during alfalfa seed germination under low temperatures (Figure 10, Supplementary Table S9), suggesting that *LACS* possibly regulated cell permeability during seed germination.

### 3.5. Glutathione Metabolism Involved in Alfalfa Seed Germination in Response to Low Temperature Stress

Glutathione is the endogenous antioxidant involved in the AsA-GSH cycle, which acts as a substrate to quench ROS and eliminate destructive peroxides [86]. Additionally, homoglutathione (hGSH), a GSH homologue, is also considered an effective antioxidant. It is thought that hGSH performs many roles that are ascribed to GSH, such as scavenging ROS, participating in xenobiotic defenses through GSH-S-transferases, and being involved in sulfur storage [87,88]. In this study, glutathione metabolism was significantly enriched in I10-6 vs. S10-6 and I10-15 vs. S10-15 (Figure 10, Supplementary Table S10), indicating that Sardi10 alfalfa had greater antioxidant capacity than Instict during seed germination under low temperature stress. Glutathione can be degraded via several enzymes, including gamma-glutamylcyclotransferase (GGCT), gamma-glutamyltranspeptidase (GCT), and glutamate-cysteine ligase (GCL). Among them, GGCT is the major one that hydrolyzes GSH to release 5-oxoproline, which is then converted to L-glutamate by 5-oxoprolinase [89]. GCL catalyzes cysteine and L-glutamate to produce L-γ-glutamylcysteine, which is subsequently converted to glutathione via the catalysis of glutathione synthetase (GSS) [86]. During alfalfa seed germination under low temperature stress, *GCL* and one *GSS* were upregulated at 6 and 15 h, and *GGCT* and *GCT* were upregulated at 15 h (Figure 10, Supplementary Table S10), demonstrating that low temperature affected glutathione metabolism.

Nicotinamide adenine dinucleotide phosphate (NADPH) is a key cofactor, and its regeneration is essential for maintaining cellular redox homeostasis to defend against oxidative stress [90]. Glucose-6-phosphate 1-dehydrogenase (G6PDH) is one of the NADPH-generating dehydrogenases, and it has been reported in maize that *ZmG6PDH1* can produce NADPH, which is used by the AsA-GSH cycle to mitigate cold oxidative damage [14]. Herein, *G6PDH* was partially induced by low temperature at 6 and 15 h of germination (Figure 10, Supplementary Table S10), indicating that *G6PDH* might contribute to eliminating ROS through the AsA-GSH cycle, thereby minimizing the damage induced by low temperature stress.

GPX is an effective ROS-scavenging enzyme, and overexpression of *Ricinus communis GPX4* enhanced the cold tolerance of seed germination in *Arabidopsis* [91]. Peroxiredoxin 6 (PRDX6) plays a key role in catalyzing the reduction of H_2_O_2_ by oxidizing GSH to form glutathione disulfide (GSSG) [92]. In addition, DHAR catalyzes dehydroascorbate to form AsA using GSH as the reducing substrate in the AsA-GSH cycle [93]. In this study, *GPX*, *DHAR*, and *PRDX6* were upregulated at 6 and 15 h of seed germination under low temperature stress, and their expression levels in Sardi10 seeds were several times as high as those in Instict seeds (Figure 10, Supplementary Table S10). These findings indicated that *GPX*, *DHAR*, and *PRDX6* contributed to reducing oxidative damage; thus, Sardi10 seeds had stronger resistance to low temperatures. Glutathione S-transferase (GST) is a versatile enzyme involved in various biological processes, such as xenobiotic detoxification, secondary metabolism, and stress responses [94]. In cucumber, as antioxidant enzyme encoding genes, *CsGSTs* were involved in cold stress and affected enzyme activities via the increased expression of their transcripts [95]. Herein, most *GSTs* were upregulated at 6 and 15 h of seed germination under low temperature stress, suggesting that the increased *GSTs* might help eliminate ROS and minimize the damage induced by low temperature in alfalfa.

## 4. Materials and Methods

### 4.1. Plant Materials

Two alfalfa cultivars of Instict (I, low temperature-sensitive genotype) and Sardi10 (S, low temperature-tolerant genotype) were selected. Seeds were harvested at maturity in 2020, with an original germination percentage of 100%. After harvest, seeds were stored in a refrigerator at −20 °C in darkness until further use.

### 4.2. Seed Germination Characteristics under Low Temperature Stress

Germination assay was performed to compare the differences in seed germination characteristics between two cultivars of alfalfa, referring to rules of International Seed Testing Association (ISTA) [96]. Three replicates of fifty uniformly plump seeds each were imbibed in a petri dish (110 mm × 110 mm), which was covered by three layers of filter paper moisturized with 10 mL of distilled water. Seeds were incubated in light chambers that were, respectively, set to 20 °C (normal temperature as control) and 10 °C (low temperature), with a photoperiod of 8 h light and 16 h darkness. Seeds were considered to germinate when radicle protruded seed coat at least 2 mm, and seedling morphogenesis was regarded as completed when normal seedlings formed without morphological defects (namely, having the normal cotyledons, hypocotyls, and roots and possessing the potential to develop into complete plants). During 10-day germination process, the number of germinated seeds and normal seedlings was monitored and recorded daily, and distilled water was regularly supplemented to prevent filter papers from drying out, thus ensuring optimal germination and growth. Then, to further quantify the phenotypic differences in seed germination between Instict and Sardi10 under low temperature stress, the related indicators, such as germination percentage, mean germination time, germination index, seed vigor index, seedling percentage, shoot length, root length, seedling fresh weight, and seedling vigor index, were calculated.

Germination percentage = (N_10_/N) × 100%

Mean germination time = ∑(N_t_ × D_t_)/∑N_t_

Germination index = ∑(N_t_/D_t_)

Seed vigor index = Germination index × Seedling fresh weight

Seedling percentage = (G_10_/N) × 100%

Seedling vigor index = (Shoot length + Root length) × Seedling percentage

N_10_ is the total number of seeds germinated in the 10 days of germination, G_10_ is the total number of normal seedlings in the 10 days of germination, N is the total number of tested seeds, N_t_ is the total number of seeds germinated on the t day, and D_t_ is the corresponding number of germination days.

### 4.3. Establishment of Seed Germination Phase under Low Temperature Stress

To determine the three phases of seed germination, dynamic changes in increased seed mass of Instict and Sardi10 during imbibition at 20 °C and 10 °C were analyzed. Briefly, for each of two alfalfa cultivars, three replicates of 100 seeds were weighed, flattened into a single layer in a petri dish covered with three layers of 10-mL distilled water-moisturized filter papers, and then imbibed, respectively, at 20 °C (normal temperature as control) and 10 °C (low temperature) in the dark. The weight of imbibed seeds was measured every 3 h, and then seed imbibition curve was established according to the increased seed mass to determine the germination phases.

### 4.4. Seed Sample Preparation

Based on seed imbibition curve, 6 h (phase I) and 15 h (phase II) were determined as the key time points for sampling during alfalfa seed germination process. Specifically, uniformly plump seeds of two alfalfa cultivars were imbibed with distilled water at 20 °C (normal temperature as control) and 10 °C (low temperature) in the dark, respectively. After imbibition for 6 and 15 h, seeds were immediately collected, frozen in liquid nitrogen, and stored at −80 °C, which were used for the subsequent transcriptome and qRT-PCR analyses. All treatments were carried out with three independent biological replicates, and samples were named as follows: Instict seeds imbibed at 20 °C for 6 h (I20-6, -1/-2/-3) and 15 h (I20-15, -1/-2/-3), Instict seeds imbibed at 10 °C for 6 h (I10-6, -1/-2/-3) and 15 h (I10-15, -1/-2/-3), Sardi10 seeds imbibed at 20 °C for 6 h (S20-6, -1/-2/-3) and 15 h (S20-15, -1/-2/-3), and Sardi10 seeds imbibed at 10 °C for 6 h (S10-6, -1/-2/-3) and 15 h (S10-15, -1/-2/-3).

### 4.5. RNA Isolation, cDNA Library Construction, and Sequencing

To investigate the key genes and potential regulatory mechanisms involved in alfalfa seed germination under low temperature stress, 24 cDNA libraries were constructed from Instict and Sardi10 seeds that germinated at normal (20 °C) and low (10 °C) temperatures. Total RNA from 24 seed samples was extracted using an RNAprep Pure Plant Kit (Tiangen, Beijing, China). RNA concentration and purity were assessed using the Nanodrop 2000 spectrophotometer (Thermo Fisher Scientific, Wilmington, DE, USA), and RNA integrity was checked on the Agilent 2100 Bioanalyzer (Agilent Technologies, Santa Clara, CA, USA). Then, mRNA was fragmented and synthesized into double-stranded cDNA, after which cDNA purification, adaptor ligation, fragment size selection, and enrichment were conducted to construct the sequencing libraries. The cDNA libraries were sequenced on an Illumina NovaSeq 6000 platform (Illumina, San Diego, CA, USA), with the sequencing mode of PE150.

### 4.6. The De Novo Assembly and Functional Annotation

Raw data in FASTQ format was processed and filtered to obtain clean reads, by removing reads with adapter pollution and low quality. The high-quality clean reads were mapped to alfalfa reference genome (Zhongmu No. 1, https://figshare.com/articles/dataset/Medicago_sativa_genome_and_annotation_files/12623960, accessed on 9 July 2020) to obtain the localization information of reads [97], using Hierarchal Indexing for Spliced Alignment of Transcripts (HISAT2, version 2.0.4) [98]. Then, the mapped reads were de novo assembled to reconstruct and identify the known and novel transcripts from HISAT2 alignment result using StringTie assembly method [99]. The functional annotation was carried out by searching against public databases of Gene Ontology (GO) [100], KEGG [101], Clusters of orthologous groups for eukaryotic complete genomes (KOG) [102], Pfam [103], Swiss-Prot [104], Evolutionary Genealogy of Genes: Non-supervised Orthologous Group (eggNOG), and Non-Redundant Protein Sequence Database (NR) [105].

### 4.7. Identification and Enrichment Analysis of Differentially Expressed Genes (DEGs)

Quantification of gene expression level was estimated by Fragments Per Kilobase of transcript per Million fragments mapped (FPKM). The differential expression analysis of each gene between pairwise comparisons of seed samples was performed using DESeq2 software (version 1.22.1), according to fold change (FC) and false discovery rate (FDR), which were controlled by adjusting *p* value through Benjamini and Hochberg’s approach. DEGs were identified using |log_2_FC| ≥ 1 and FDR < 0.01 as screening thresholds. GO functional enrichment and KEGG pathway enrichment analyses of DEGs were, respectively, implemented by ClusterProfiler packages based on Wallenius non-central hyper-geometric distribution [106] and KOBAS software (version 3.0) [107], with a significance level of *p* value < 0.05.

### 4.8. WGCNA Analysis

RNA-seq data were analyzed to construct the gene co-expression network using the WGCNA R package (version 4.2.2) [108], with program parameters of depth split = 1, minimum module size = 30, and merge cut height = 2. Module eigengenes were the first major component of expression matrix, which summarized the module overview and feature data. Pearson’s correlation coefficient was used to calculate the correlation between modular eigengenes and treatments of two alfalfa cultivars under different temperatures and imbibition durations. The correlation was reflected by the color depth in heatmap. Cytoscape software (version 3.7.1) was used for drawing the regulatory network and highly significant modules with particular WGCNA edge weight [109].

### 4.9. Validation of Key Candidate DEGs by qRT-PCR

To verify the reliability of transcriptomic data, eleven DEGs involved in seed germination responses to low temperatures were selected for qRT-PCR. The same RNA samples for transcriptome sequencing were reverse transcribed to cDNA using a HiScript III 1st Strand cDNA Synthesis Kit (+gDNA wiper) (R312-02, Vazyme, Nanjing, China). The qRT-PCR reaction system contained 10 µL of ChamQ SYBR Color qPCR Master Mix (Q411-2, Vazyme, Nanjing, China), 0.4 µL of forward primer, 0.4 µL of reverse primer, 2 µL of cDNA, and 7.2 µL of ddH_2_O, reaching a final volume of 20 µL. The qRT-PCR analysis was carried out on a CFX96 TOUCH instrument (Bio-rad, Hercules, CA, USA) with the following program: 95 °C for 30 s, followed by 40 cycles at 95 °C for 10 s, 60 °C for 30 s, and 72 °C for 30 s. All samples were examined in three biological replicates and three technological replicates. *MsActin* was used as the reference gene. Relative expression levels of selected DEGs were calculated via the 2^−ΔΔCt^ method [110]. Primers were designed using Primer (version 5.0), and the sequences are displayed in Supplementary Table S11.

### 4.10. Statistical Analysis

All statistical analyses were performed using SPSS software (SPSS Inc., Chicago, IL, USA, version 25.0). For seed germination data, values were expressed as means ± SE (n = 3). Duncan’s test, or Student’s *t*-test, was applied to evaluate the difference among treatments at a 5% significant level. Graphics were drawn using the GraphPad Prism software (GraphPad Inc., La Jolla, CA, USA, version 9.5).

## 5. Conclusions

In general, comparative phenotypic and transcriptomic analyses were performed on the seeds of two alfalfa cultivars that presented different germination performance under low temperature stress, and the possible mechanisms and key biological pathways involved in low temperature tolerance were preliminary revealed (Figure 10). Transcriptome analyses successfully identified four regulatory pathways involved in alfalfa seed germination under low temperature stress. Among them, galactose metabolism and carbon metabolism pathways were significantly enriched in both two alfalfa cultivars at 6 and 15 h of germination under low temperature stress. Furthermore, the preferred pathways of fatty acid metabolism and glutathione metabolism were significantly enriched in Sardi10. These pathways and the involved DEGs might be conducive to explaining why Sardi10 alfalfa had a stronger low temperature tolerance and higher germination capacity than Instict, which were manifested as the relatively rapid water uptake, activation process, and root/seedling development. It was also found that the expression levels of key candidate genes such as *GPX*, *DHAR*, *GST*, *PEPC*, *ICL*, *GolS*, *RAFS*, *α-Gal*, and *accB* were significantly affected by low temperature stress. Summarily, these findings suggested that the alterations in galactose metabolism, carbon metabolism, fatty acid metabolism, and glutathione metabolism might be closely related to alfalfa seed germination affected by low temperatures. This study provides new insights into alfalfa seed germination in response to low temperature stress. In the future, single-cell RNA sequencing, spatial transcriptomics, or stereo-seq can be used to elucidate the cell type for each gene expression difference during alfalfa seed germination under low temperature stress.

## Data Availability

Data supporting the results are contained within the article and Supplementary Materials. The transcriptome raw data presented in this study can be found in online repositories. The names of the repositories and accession numbers can be found below: NCBI SRA BioProject with accession number of PRJNA1125405.

## Supplementary Materials

The following supporting information can be downloaded at: https://www.mdpi.com/article/10.3390/ijms25137244/s1.
